# Proton Pump Inhibitors Use and Risk of Preeclampsia: A Meta-Analysis

**DOI:** 10.3390/jcm11164675

**Published:** 2022-08-10

**Authors:** Salman Hussain, Ambrish Singh, Benny Antony, Jitka Klugarová, M. Hassan Murad, Aarthi S. Jayraj, Alena Langaufová, Miloslav Klugar

**Affiliations:** 1 Czech National Centre for Evidence-Based Healthcare and Knowledge Translation (Cochrane Czech Republic, Czech EBHC: JBI Centre of Excellence, Masaryk University GRADE Centre), Institute of Biostatistics and Analyses, Faculty of Medicine, Masaryk University, Brno, Czech Republic, Kamenice 5, 62500 Brno, Czech Republic; 2 Menzies Institute for Medical Research, University of Tasmania, 17 Liverpool St, Hobart, TAS 7000, Australia; 3 Division of Public Health, Infectious Diseases and Occupational Medicine, Mayo Clinic, 200 1st St SW, Rochester, MN 55905, USA; 4 Department of Obstetrics & Gynaecology, All India Institute of Medical Sciences, New Delhi 110029, India

**Keywords:** hypertension, preeclampsia, proton pump inhibitors, PPIs, pregnancy, meta-analysis

## Abstract

Evidence from preclinical studies suggests a preventive effect of proton pump inhibitors (PPIs) in preeclampsia. Recently, several epidemiological studies have described a conflicting association between the use of PPIs during pregnancy and preeclampsia risk. This study aimed to evaluate the association between PPI use and the risk of preeclampsia. We searched databases, including MEDLINE, Embase, Scopus, Web of Science Core Collection, Emcare, CINAHL, and the relevant grey literature from inception until 13 September 2021. Studies reporting the preeclampsia risk with the use of PPIs were eligible for inclusion. Literature screening, data extraction, and the risk of bias assessment were performed independently by two investigators. Random-effect meta-analysis was performed to generate relative risks (RR) and 95% confidence intervals (CI). The risk of preeclampsia and preterm preeclampsia among women receiving PPIs during pregnancy were the primary outcomes of interest. This meta-analysis comprised three studies involving 4,877,565 pregnant women, of whom 119,017 were PPI users. The included studies were judged to have a low risk of bias. The risk of preeclampsia among pregnant women who received PPIs anytime during pregnancy was significantly increased (RR 1.27 (95% CI: 1.23–1.31)), although the increase was trivial in absolute terms (2 per 1000). The subgroup analysis revealed that the risk was increased in each of the three trimesters. The risk of preterm preeclampsia among pregnant women receiving PPIs anytime during pregnancy was not significantly increased (RR 1.04 (95% CI: 0.70–1.55)). The certainty evaluated by GRADE in these estimates was low. PPI use may be associated with a trivial increase in the risk of preeclampsia in pregnant women. There is no evidence supporting that PPI use decreases the risk of preeclampsia or preterm preeclampsia.

## 1. Introduction

Preeclampsia is one of the most severe complications of pregnancy characterized by high blood pressure. It is one of the leading causes of maternal morbidity and mortality worldwide. The global burden of preeclampsia is continuously rising; epidemiological trends showed a 10.9% increase in the incidence of preeclampsia from 1990 to 2019 [1]. Preeclampsia leads to adverse maternal and perinatal outcomes, including preterm birth, prolonged hospital stays, low birthweight babies, and a higher risk of neonatal intensive care unit admission [2]. Preterm birth imposes a significant mortality risk on the mother and the baby [3]. Several database studies have reported a positive association between preterm birth and mortality and morbidity in both the mother and the baby [4,5]. There are no definitive treatment options available for preeclampsia management, except for the timely delivery of the fetus and placenta.

Evidence from preclinical studies suggests a plausible preventive effect of proton pump inhibitors (PPIs) in preeclampsia [6,7,8]. The potential mechanism of this protective effect of PPIs in managing preeclampsia could be due to the reduction in the mRNA expression and secretion of antiangiogenic factors (sFlt1) and soluble endoglin (sEng) in placental endothelial cells, as these are the key component involved in the pathophysiology of preeclampsia [7,9,10]. This mechanistic association was supported by the findings of a recently published prospective cohort study where lower levels of sFLT-1 and sEng were noticed among pregnant PPI users with suspected preeclampsia [11]. PPIs are commonly used to treat gastroesophageal reflux disorder (GERD); however, in the last decade, the safety of PPIs has been a matter of scrutiny [12]. Our previous systematic reviews and meta-analyses found PPI use to be associated with several other non-pregnancy-related adverse health outcomes [13,14,15,16]. Nevertheless, PPIs are widely used by pregnant women due to their acceptable safety profiles and their availability as over-the-counter (OTC) drugs in many countries [17]. The evidence from cohort studies and a meta-analysis supported the PPI safety profiles among women who used PPIs during pregnancy and found no increased risk of congenital defects or preterm delivery [18,19].

Recently, several epidemiological studies examined the association of PPIs with preeclampsia risk [20,21,22]. A large cohort study from the US using the Truven Health MarketScan database found no association of PPIs with a decreased risk of preeclampsia or severe preterm preeclampsia [22]. Similar findings were reported by Choi et al. using the Korean Healthcare database [21]. However, a Swedish population register-based cohort study found reduced preterm and early preeclampsia risk in women who used PPIs in the third trimester [20]. These published studies presented conflicting evidence, and to date, no meta-analysis has been performed to explore this association, as confirmed through a preliminary search in multiple databases. Therefore, this systematic review and meta-analysis aimed to synthesize the evidence and assess the overall risk of preeclampsia in women using PPIs during pregnancy.

## 2. Materials and Methods

### 2.1. Protocol

The protocol for this systematic review was prospectively published as a preprint at medRxiv [23]. The principles laid down in the Cochrane Handbook of Systematic Review of Interventions and the JBI reviewers manual were utilized [24,25]. Preferred reporting items for systematic review and meta-analysis (PRISMA 2020) and meta-analysis of observational studies in epidemiology (MOOSE) reporting guidelines were followed [26,27]. Refer to Supplementary Table S1 for the detailed checklist.

### 2.2. Search Strategy

The literature search was conducted in each database from the inception date to 13 September 2021 to identify published and unpublished studies assessing preeclampsia risk in women receiving PPIs during pregnancy. The three-step search strategy was used; an initial limited search was conducted in MEDLINE (Ovid) and Embase (Ovid), using keywords and index terms related to PPIs and preeclampsia without restriction to any date or language; then, a detailed search was performed across all major databases by analyzing the text words and index terms used to describe the articles. We searched MEDLINE (Ovid), Embase (Ovid), Scopus, Web of Science Core Collection, Emcare (Ovand id), and CINAHL (EBSCO). The sources of grey literature were ProQuest Dissertations & Theses Global and clinical trials registers, ClinicalTrials.gov (accessed on 13 September 2021), and the WHO International Clinical Trials Registry Platform (ICTRP). Search strings were developed by a medical information specialist (AL). Lastly, bibliographies of the relevant articles were scanned manually for additional articles. The search strategies used for different databases were provided in Supplementary Table S2 with their respective hits.

### 2.3. Study Selection/Inclusion Criteria

Two reviewers independently reviewed the retrieved articles based on title and abstract screening, which was followed by second-level screening based on full-text articles. Covidence systematic review software was used for completing the article screening process [28].

Studies were eligible for inclusion if they met the inclusion criteria mentioned in Table 1:

### 2.4. Data Extraction and Risk of Bias

Two reviewers independently extracted all the relevant data based on the study characteristics (author, publication year, data source, and study period); details on patient characteristics; exposure; comparator; ascertainment of PPI use; confirmation of outcome; effect estimates (unadjusted and adjusted risk); and conclusions. Any discrepancy during the data extraction process was resolved by discussion. There was no missing data, so none of the primary authors was contacted for any additional data.

The risk of bias in the included studies was evaluated using the Newcastle–Ottawa Scale (NOS) independently by two reviewers [29]. According to the NOS, a study can achieve a maximum of 4 points in the selection, 2 points in the comparability, and 3 points in the exposure (case–control studies) or outcome (cohort studies) domain of the scale. Studies were classified as having a high, moderate, or low risk of bias, depending on the adjustment for appropriate confounders and the adequacy of the exposure and outcome ascertainment and not based on a numerical score [30].

### 2.5. Certainty of Evidence

We used the Grading of Recommendations Assessment, Development and Evaluation (GRADE) methodology to assess the certainty of evidence [31]. The certainty assessment was judged as either high, moderate, low, or very low, based on the risk of bias, inconsistency, indirectness, imprecision, and publication bias.

### 2.6. Statistical Analysis

The primary outcome of interest was to assess the pooled relative risk of preeclampsia among women receiving PPIs during pregnancy. The risk ratio (RR) and odds ratio were used interchangeably, as PPI use and preeclampsia events were very rare [32]. Heterogeneity was determined based on Cochrane chi-square and I2 statistics [33]. The Cochrane chi-square value (*p* < 0.10) and I2 statistics ≥ 50% represent important heterogeneity [33]. Since we anticipated heterogeneity in terms of the population characteristics and settings of the studies, we used the random effect model. A subgroup analysis was performed based on the trimester of pregnancy and preterm and term preeclampsia. The leave-one-out method was used to understand the impact of each study on the pooled effect size. Meta-regression and statistical evaluation of the publication bias using funnel plot approaches were not feasible due to the limited number of studies. Review Manager (RevMan) version 5.4.1 was used to perform the meta-analysis using the generic inverse variance method. Summary of the findings table was created using the GRADEpro GDT tool [34].

## 3. Results

### 3.1. Studies Characteristics

**Figure 1:**
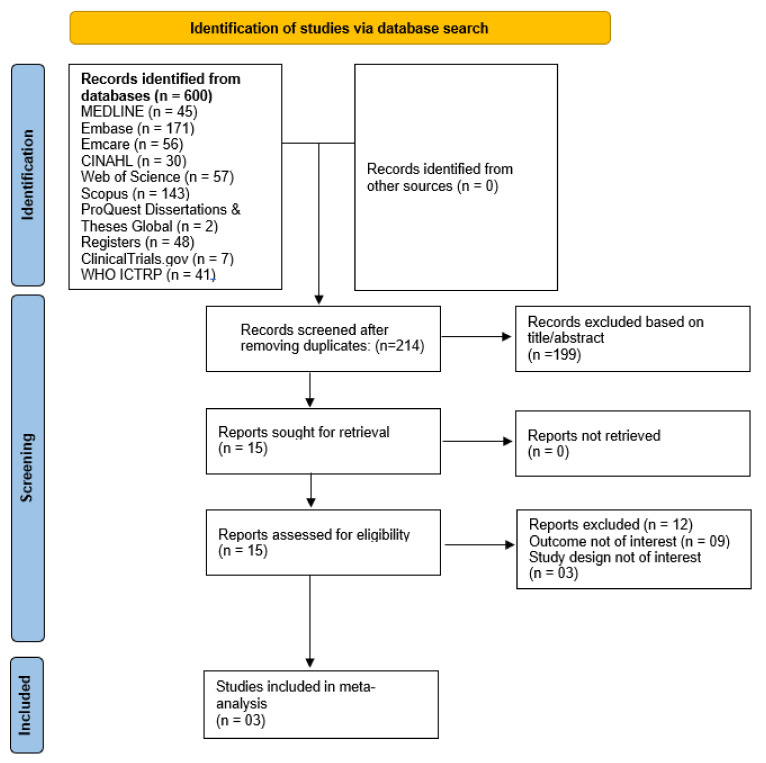
PRISMA flowchart showing the study inclusion process.

The database search yielded 600 articles; three studies [20,21,22] involving 4,877,565 pregnant women, of whom 119,017 were PPI users, were included in the meta-analysis. A list of articles excluded with exclusion reasons during the full-text screening phase are presented in Supplementary Table S3. Refer to the PRISMA chart (Figure 1) for the study inclusion process.

The design of the three studies was a retrospective cohort design, and they were published within the time frame of 2019–2021. All the eligible studies were published as a full text, except the study by Choi et al., published as a research letter. Included studies were conducted in the US, Sweden, and Korea. PPI exposure was defined as exposure to PPIs at any time during pregnancy or individually during the first, second, and third trimesters. Studies ascertained the PPI exposure through prescription records or claims data, and the outcome of preeclampsia was confirmed based on the International Classification of Diseases (ICD) code—9th or 10th edition. Refer to Table 2 for a detailed description of the included study characteristics.

### 3.2. Quality Assessment and Certainty of Evidence

The risk of bias in the three included studies was low. All the included studies selected the patients and control from the same databases and adjusted for several possible confounding factors. The details of this assessment are presented in Table 3. The certainty of the evidence on the risk of preeclampsia and preterm preeclampsia among women receiving PPIs during pregnancy was low to very low, as per the GRADE rating system (Table 4a,b). Of note, the absolute effects were trivial or very small.

### 3.3. Meta-Analysis (Preeclampsia Risk)

**Figure 2:**
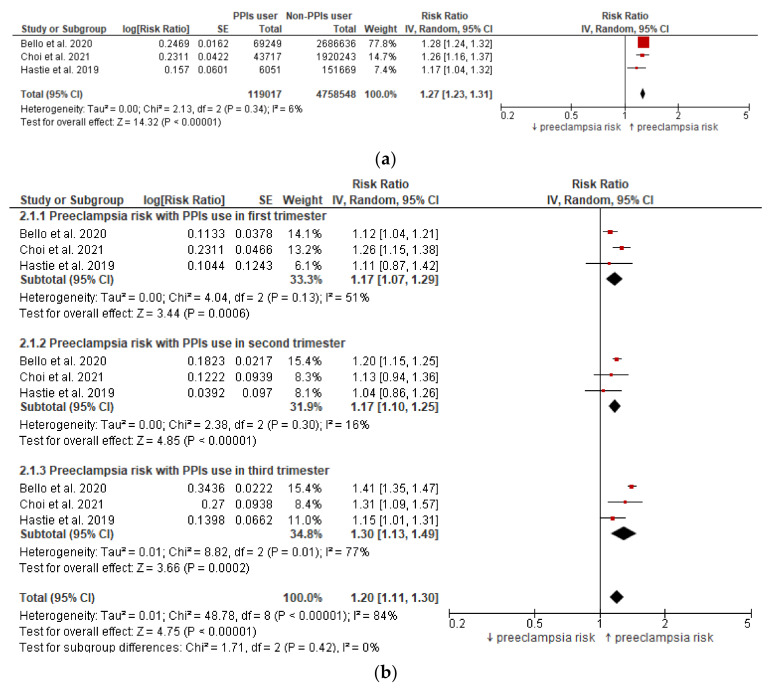
(**a**) Preeclampsia risk in women exposed to PPIs anytime during pregnancy. (**b**) Preeclampsia risk in women exposed to PPIs in different trimesters of pregnancy.

The risk of preeclampsia among pregnant women who received PPIs anytime during pregnancy was statistically significantly increased (Figure 2a) with a pooled RR of 1.27 (95% CI: 1.23–1.31), *p* < 0.00001 in an adjusted analysis (adjusted for several possible confounding factors).

The subgroup analysis revealed a statistically significantly higher risk of preeclampsia in pregnant women receiving PPIs in each of the three trimesters (Figure 2b). All the analyses were adjusted for maternal age, chronic kidney disease, autoimmune disease, multiple gestation, pregestational diabetes, chronic hypertension, nulliparity, and multiple pregnancies.

### 3.4. Meta-Analysis (Preterm Preeclampsia Risk)

**Figure 3:**
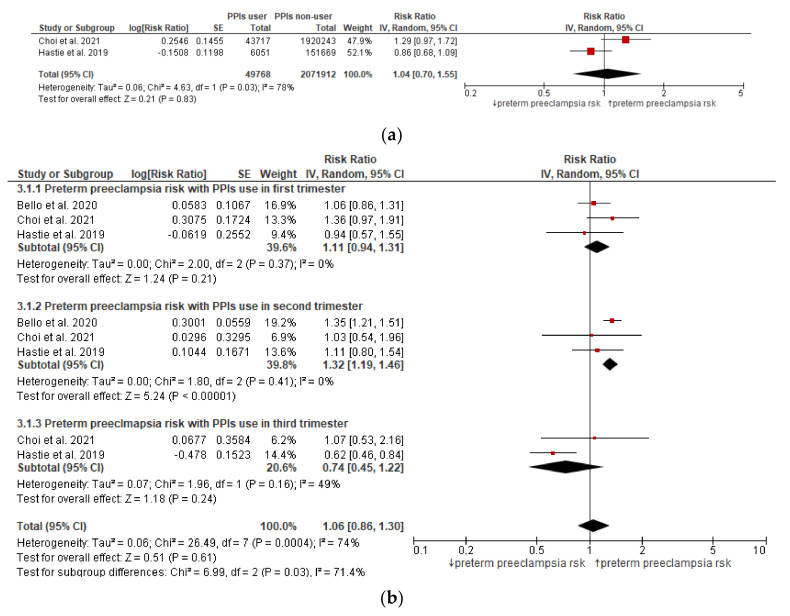
(**a**) Preterm preeclampsia risk in women exposed to PPIs anytime during pregnancy. (**b**) Preterm preeclampsia risk in women exposed to PPIs in different trimesters of pregnancy.

Two studies reported data for the preterm preeclampsia risk among pregnant women receiving PPIs anytime during pregnancy, and the pooled estimate (Figure 3a) revealed a nonsignificant association (*p* = 0.83).

The subgroup analysis based on the use of PPIs in various trimesters and the risk of preterm preeclampsia (Figure 3b) revealed a significantly higher risk in the second trimester, with a pooled relative risk of 1.32 (95% CI: 1.19–1.46), *p* < 0.00001. However, the association was nonsignificant in the first or third trimesters.

### 3.5. Sensitivity Analysis

A sensitivity analysis was performed by excluding each study one by one (leave-one-out) from the pooled analysis. None of the studies had any significant influence on the pooled effect size. The RR for preeclampsia was identical to the main results.

## 4. Discussion

This is the first systematic review and meta-analysis to investigate the risk of preeclampsia and preterm preeclampsia among women receiving PPIs during pregnancy. We found a significantly higher risk of preeclampsia in pregnant women exposed to PPIs anytime during pregnancy or during each specific trimester, although this risk was trivial or very small in absolute terms. PPI use was also associated with a significant increase in the risk of preterm preeclampsia only in the second trimester.

The certainty in the estimates was low, which suggests the need for randomized trials that evaluate patient important outcomes, as very few trials exist or are ongoing. One trial has demonstrated no prolongation in the gestation period with esomeprazole in women with preterm preeclampsia [35]. In addition, that trial showed no significant change in the relevant biomarkers levels (sFlt1, sEng, and placental growth factor) and maternal, fetal, or neonatal outcomes [35]. Similarly, a recently published randomized, double-blinded placebo-controlled trial by Abbas et al. [36] also confirmed no significant change in the antiangiogenic markers in women with early onset preeclampsia who received 40 mg of esomeprazole daily. This trial also found a nonsignificant effect of esomeprazole on the length of pregnancy and maternal and fetal complications [36]. Several trials are currently underway to assess the efficacy of PPIs, either alone or in combination with agents such as metformin or sildenafil for the management of preeclampsia. Table 5 provides a detailed list of the relevant ongoing trials.

The strength of the current systematic review was the exhaustive search for all the eligible published and unpublished studies from multiple literature sources, including gray literature, enabling a large sample size to estimate the risk of both preeclampsia and preterm preeclampsia based on the use of PPIs in different gestation ages.

However, a few important limitations need to be considered. First, the included studies did not specifically ascertain medication adherence, and the assessment of PPI exposure was based on the claims data and prescription records. Further, the availability of PPIs as OTC drugs in two of the included studies may lead to an inaccurate assessment of their exposure and, thus, raise the possibility of confounding [37]. Second, only one study adjusted the findings for the body mass index (BMI), although the BMI is known to be associated with preeclampsia [38]. Lastly, a small number of studies and the observational nature of the included studies and confounding by indication should be considered while interpreting the findings.

## 5. Conclusions

PPIs use may be associated with a trivial increase in the risk of preeclampsia in pregnant women. There is no evidence supporting that PPI use decreases the risk of preeclampsia or preterm preeclampsia. We recommend that future epidemiological studies consider all possible confounding factors, including the BMI. Furthermore, future population-based studies should ascertain the risk of preeclampsia and preterm preeclampsia separately by including a sufficiently large number of (preeclampsia and preterm preeclampsia) cases. The ongoing clinical trials of PPIs are expected to shed light on this important clinical question.

## Figures and Tables

**Table 1:** Eligibility criteria for the selection of articles.

Criterion	Inclusion Criteria	Exclusion Criteria
Population	Pregnant women at any stage of gestation	Non-pregnant women
Exposure	Exposure to any proton pump inhibitors Omeprazole Esomeprazole Pantoprazole Rabeprazole Lansoprazole Dexlansoprazole Ilaprazole	Drugs other than proton pump inhibitors
Comparator	Nonexposure or exposure to H2RA antagonist	N/A
Outcomes	Studies reporting: Preeclampsia risk at any stage of pregnancy Preterm preeclampsia risk	Studies reporting any other outcomes: Cost-effectiveness HRQoL Cost and resource use
Study design	Studies assessing preeclampsia risk, including: Retrospective cohort Prospective cohort Case–control studies	Following was excluded: Animal studies In vitro studies Literature reviews Pharmacodynamic and pharmacokinetic studies
Time period	Studies published until September 2021	N/A

H2RA: Histamine 2 receptors antagonist; HRQoL: Health-related quality of life; N/A: Not applicable.

**Table 2:** Characteristics of the included studies.

Author, Year & Country	Study Design, Setting	Study Duration	Database/Source	Participants	Exposure	Comparator	Outcomes	Cohort Size	Definition of PPI Exposure	Ascertainment of PPI Use	Assessment of Outcome	Effect Estimates		Adjusted for	Conclusion
												Unadjusted	Adjusted		
Bello et al., 2020, US [22]	Cohort study	2008 to 2014	Truven Health MarketScan database	Women receiving PPIs during pregnancy in the Truven Health MarketScan Database	PPI user (Esomeprazole, lansoprazole, omeprazole, pantoprazole, dexlansoprazole, and rabeprazole)	No exposure to PPIs	Diagnosis of preecalmpsia	Total: 2,755,885 PPI user: 69,249 Non-PPI user: 2,686,636	PPI exposure any time during pregnancy or individually during the 1st, 2nd, and 3rd trimesters	Outpatient pharmaceutical claims data	Idiopathic PD diagnosis confirmed by based on the presence of International Classification of Diseases, Ninth Edition, Clinical Modification (ICD-9-CM) diagnosis codes for mild (642.4×), severe (642.5×), or superimposed (642.7×) preeclampsia or eclampsia (642.6×).	**Preeclampsia** Any time PPI use: 1.42 (1.38, 1.46) 1st trimester PPI use: 1.20 (1.11, 1.30) 2nd trimester PPI use: 1.34 (1.28, 1.41) 3rd trimester PPI use: 1.56 (1.50, 1.63) **Preterm severe preeclampsia/** **Eclampsia** 1st trimester PPI use: 1.15 (0.93, 1.43) 2nd trimester PPI use: 1.58 (1.41, 1.77)	**Preeclampsia** Any time PPI use: 1.28 (1.24, 1.32) 1st trimester PPI use: 1.12 (1.04, 1.22) 2nd trimester PPI use: 1.20 (1.15, 1.26) 3rd trimester PPI use: 1.41 (1.35, 1.47) **Preterm severe preeclampsia/** **Eclampsia** 1st trimester PPI use: 1.06 (0.86, 1.32) 2nd trimester PPI use: 1.35 (1.21, 1.52)	Maternal age, and the five clinical characteristics (chronic kidney disease, autoimmune disease, multiple gestation, Pregestational diabetes, and chronic hypertension)	PPI prescription during pregnancy was not associated with decreased risk for preeclampsia
Choi et al., 2021, Korea [21]	Cohort study	2011 to 2017	Health Insurance Review and Assessment database	Women receiving PPIs during pregnancy in Korea’s healthcare database	Use of any PPI, including omeprazole, esomeprazole, pantoprazole, rabeprazole, lansoprazole, dexlansoprazole, or ilaprazole at any point across gestation	(1). Non-PPI user, and (2). H2RA user	Diagnosis of preecalmpsia	Total: 1,963,960 PPI user: 43,717 Non-PPI user: 1,920,243	≥1 PPI prescription in 4 windows: any time during pregnancy, first, second, and third trimester	Database (based on drug chemical code, prescription supply, dosage, and others)	ICD-10 diagnostic code	**Preeclampsia** Any time PPI use: 1.55 (1.44–1.68) 1st trimester PPI use: 1.56 (1.42, 1.72) 2nd trimester PPI use: 1.43 (1.19, 1.71) 3rd trimester PPI use: 1.69 (1.42, 2.03) **Preterm preeclampsia** Any time PPI use: 1.55 (1.18–2.04) 1st trimester PPI use: 1.62 (1.17–2.24) 2nd trimester PPI use: 1.31 (0.68–2.52) 3rd trimester PPI use: 1.37 (0.68–2.74)	**Preeclampsia** Any time PPI use: 1.26 (1.16–1.36) 1st trimester PPI use: 1.26 (1.15, 1.39) 2nd trimester PPI use: 1.13 (0.94, 1.35) **3rd trimester PPI use:** 1.31 (1.09, 1.56) **Preterm preeclampsia ** Any time PPI use: 1.29 (0.97–1.71) 1st trimester PPI use: 1.36 (0.97–1.89) 2nd trimester PPI use: 1.03 (0.54–1.99) 3rd trimester PPI use: 1.07 (0.53–2.14)	Maternal age and insurance type, nulliparity, multiple gestation, CCI, indications for acid suppressive medications, including gastroesophageal reflux disease, heartburn, ulcer (e.g., various ulcers and ZES), maternal medical conditions (e.g., asthma, anxiety, diabetes, depression, and chronic hypertension), inflammatory diseases, migraine/headache, renal disease, thyroid disorder, concurrent medications, and proxies of health care utilization	PPI use during pregnancy was not associated with a reduced risk of preeclampsia
Hastie et al., 2019, Sweden [20]	Cohort study	2013 to 2017	Swedish pregnancy register	Women receiving PPIs during pregnancy in Swedish pregnancy register	Use of any PPI, including omeprazole, esomeprazole, pantoprazole, rabeprazole, or lansoprazole at any point across gestation	Non-PPI users	Diagnosis of preecalmpsia	Total: 157,720 PPI user: 6051 Non-PPI user: 151,669	PPI use was categorized by use ever during pregnancy, first trimester (0–12 weeks of gestation), second trimester (13–27 weeks), and third trimester (from 28 weeks of gestation onward).	Based on the prescription record maintained in Swedish pregnancy register	Preeclampsia was identified by the diagnosis codes O14 or O15 according to International Classification of Diseases, Tenth Revision coding (n = 7258)	**Preeclampsia ** Any time PPI use: 1.20 (1.07–1.35) 1st trimester PPI use: 1.20 (0.95, 1.52) 2nd trimester PPI use: 1.15 (0.97, 1.36) 3rd trimester PPI use: 1.21 (1.07, 1.37) **Preterm preeclampsia ** Any time PPI use: 0.90 (0.71–1.13) 1st trimester PPI use: 0.95 (0.59–1.49) 2nd trimester PPI use: 1.13(0.83–1.54) 3rd trimester PPI use: 0.66 (0.40–1.07)	**Preeclampsia ** Any time PPI use: 1.17 (1.04–1.32) 1st trimester PPI use: 1.11 (0.87–1.42) 2nd trimester PPI use: 1.04 (0.86–1.25) 3rd trimester PPI use: 1.15 (1.01–1.32) **Preterm preeclampsia **Any time PPI use: 0.86 (0.68–1.09) 1st trimester PPI use: 0.94 (0.57–1.54) 2nd trimester PPI use: 1.11(0.80–1.54) 3rd trimester PPI use: 0.62 (0.46–0.84)	Propensity matched (maternal age, body mass index, year of delivery, country of birth, smoking status, educational level, occupation, use of assisted reproduction, and the presence of pregestational disorders	PPIs have a potential role in preventing preterm preeclampsia

CCI: Charlson comorbidity index; H2RA: histamine 2 receptor antagonists; PPI: proton pump inhibitor; ZES: Zollinger-Ellison syndrome.

**Table 3:** Quality assessment of the included studies.

Cohort Studies	Selection				Comparability	Outcome			
Study author	Representation of the exposed cohort	Selection of the non-exposed cohort	Ascertainment of exposure	Demonstration that outcome of interest was not present at the start of the study	Comparability of cohorts on the basis of design or analysis	Assessment of outcome	Was follow-up long enough for outcomes to occur	Accuracy of follow-up of cohorts	Overall risk of bias
Bello, 2020, US [22]	*	*	*	*	**	*	*	*	Low
Choi, 2021, Korea [21]	*	*	*	*	**	*	*	*	Low
Hastie, 2019, Sweden [20]	*	*	*	*	**	*	*	*	Low

* = this symbol represents the number of stars given to each category according to the star-based scoring systems employed to assess the risk of bias of each study as detailed in the Section 2.4 in the main text.

**Table 4:** (**a**) Summary of the findings table showing the certainty of the evidence for preeclampsia risk in women exposed to PPIs (anytime during pregnancy) compared to non-PPIs. (**b**) Summary of the findings table showing the certainty of the evidence for preterm preeclampsia risk in women exposed to PPIs (anytime during pregnancy) compared to non-PPIs.

Certainty Assessment							№ of Patients		Effect		Certainty
№ of Studies	Study Design	Risk of Bias	Inconsistency	Indirectness	Imprecision	Other Considerations	Preeclampsia during AnyTime PPIs Use	Placebo	Relative (95% CI)	Absolute (95% CI)	
**Preeclampsia risk**											
3	observational studies	not serious	not serious	not serious	not serious	none	1294/119,017 (1.1%)	31,204/4,758,548 (0.7%)	**RR 1.27** (1.23 to 1.31)	**2 more per 1000** (from 2 more to 2 more)	⨁⨁◯◯ Low
**Certainty assessment**							**№ of patients**		**Effect**		**Certainty**
**№ of Studies**	**Study Design**	**Risk of Bias**	**Inconsistency**	**Indirectness**	**Imprecision**	**Other Considerations**	**Preterm preeclampsia during Anytime PPIs Use**	**Placebo**	**Relative (95% CI)**	**Absolute (95% CI)**	
**Preterm preeclampsia risk**											
2	observational studies	not serious	serious ^a^	not serious	not serious	none	129/49,768 (0.3%)	3626/2,071,912(0.2%)	**RR 1.04** (0.70–1.55)	**0 fewer per 1000** (from 1 fewer to 1 more)	⨁◯◯◯ VERY Low

(**a**) Patients: Preeclampsia risk in pregnant women exposed to PPIs compared to non-PPIs. Risk factor: PPI. Comparisons: Non-PPI user. CI: Confidence interval; RR: Risk ratio. GRADE Working Group grades of evidence: Low certainty: The true effect might be markedly different from the estimated effect. (**b**) Patients: Preterm preeclampsia risk in pregnant women exposed to PPIs compared to non-PPIs. Risk factor: PPI. Comparisons: Non-PPI user. CI: Confidence interval; RR: Risk ratio. Explanations: ^a^ High heterogeneity was observed with an I^2^ value of 78%. GRADE Working Group grades of evidence: Very low certainty. The true effect is probably markedly different from the estimated effect.

**Table 5:** List of ongoing clinical trials.

Trial Number	Trial Name or Title	Methods	Participants	Interventions	Outcomes	Start Date	Recruitment Status	Link to Trials
NCT03717740	Esomeprazole for the Prevention of Preeclampsia	Randomized double-blinded placebo-controlled intervention trial	Pregnant women presenting prior to 17 + 0 weeks’ gestation with moderate to high risk of preeclampsia	Esomeprazole single dose of 40 mg orally once a day from 12+ and 17 weeks of pregnancy until 34 weeks of pregnancy	Primary Outcome Measures: Number of Participants With early onset Preeclampsia Secondary Outcome Measures: Prevention of preeclampsia between 37 and 41 The number of cases of Fetal Growth Restriction The number of cases of preterm birth	1 December 2018	Recruiting	https://clinicaltrials.gov/ct2/show/NCT03717740 (accessed on 4 October 2021)
NCT03717701	Metformin and Esomeprazole in Treatment of Early Onset Preeclampsia	Randomized double-blinded placebo-controlled intervention trial	Pregnant women presenting at a Gestational age between 28 + 0 weeks and 32 + 0 weeks presented with preterm preeclampsia	Metformin 1000 mg orally once a day; Esmoperazole 40 mg orally once a day	Primary Outcome Measures: Prolongation of gestation measured from the time of enrollment to the time of delivery. Secondary Outcome Measures: Severe morbidity The change in serum level of sFlt-1 and endoglin Any side effects	1 December 2018	Recruiting	https://clinicaltrials.gov/ct2/show/NCT03717701 (accessed on 4 October 2021)
NCT03724838	Esomeprazole With Sildenafil Citrate in Women With Early-onset Preeclampsia	Randomized, double-blind, placebo-controlled trial	Pregnant women presenting at a Gestational age between 28 + 0 weeks and 32 + 0 weeks presented with preterm preeclampsia	Patients will take esomeprazole single dose of 40 mg orally once a day; Patients will take Sildenafil Citrate 40 mg every 8 h; other comparators	Primary outcome measures: Prolongation of gestation measured from the time of enrollment to the time of delivery Secondary outcome measures: Severe morbidity Side effects The change in serum level of sFlt-1 and endoglin	1 December 2018	Recruiting	https://clinicaltrials.gov/ct2/show/NCT03724838 (accessed on 4 October 2021)
EUCTR2018-000283-28-NL or Netherland Trial Register L7718	Potential effect of proton-pump inhibitor on angiogenic markers in preeclampsia: a pilot study	Randomised controlled trial	Women with (≥18 years) with a singleton pregnancy diagnosed with PE with a gestational age of ≥20 weeks and <34 weeks	Omeprazole	Primary outcome measures: The difference in sFlt-1 levels in women who have received PPI, in comparison to women who have not received PPI, at different time points. Secondary outcome measures: The change in serum levels of PlGF, sEndoglin, ET-1 and CT-proET-1 levels between PPI and non-PPI group at different time points (before and after administration) The change in cord blood levels of sFlt-1, PlGF, sEndoglin, ET-1 and CT-proET-1 at time of delivery between PPI and non-PPI group.	17 December 2018	Ongoing	https://www.clinicaltrialsregister.eu/ctr-search/trial/2018-000283-28/NL; (accessed on 4 October 2021) https://www.trialregister.nl/trial/7718 (accessed on 4 October 2021)
IRCT2017082333680N2	The evaluation of esomeprazole efficacy in treatment of early onset pre- eclampsia	Randomized, single-blind, placebo-controlled trial	Pregnant women with hypertensive Pregnancy and the gestational age between 26 to 32 weeks with single-crowned pregnanc	The intervention group received 12 mg Betamethasone in two doses every 24 h plus prescribed 40 mg osmoparazole daily. The control group received 12 mg Betamethasone in two doses every 24 h plus prescribed 40 mg placebo daily.	Primary outcome measure: Duration of admission to delivery Secondary outcome measure: Frequency of maternal and fetal complications in patients with preeclampsia Biomarker level of tyrosine kinase and endoglycine	18 April 2017	Ongoing	https://en.irct.ir/trial/25917 (accessed on 4 October 2021)
ChiCTR1900026972	A randomized controlled trial for efficacy of esomeprazole in the treatment of early-onset preeclampsia	Randomized controlled trial	Pregnant women with gestational age between 26 + 0 weeks and 33+ 6 weeks; Diagnosis of pre-eclampsia, gestational hypertension	Forty milligrams of esomeprazole+ Standard treatment vs. control group	Primary outcome measure: Duration of admission to delivery Secondary outcome measures: The change in levels of sFlt-1, and sEndoglin	1 January 2020	Recruiting	https://www.chictr.org.cn/showprojen.aspx?proj=44939 (accessed on 4 October 2021)
ACTRN12618000690257	A Prospective, Pre-ecLampsia/Eclampsia Prevention IntervEntion	Multi-centre, double blind, randomised, placebo-controlled trial	Nulliparous women with singleton pregnancy (12–20 weeks)	Forty milligrams of oral esomeprazole tablets once daily	Primary outcome measure: Incidence of preeclampsia Secondary outcome measure: Incidence of term preeclampsia Gestation of pregnancy at delivery fetal/neonatal complications/adverse outcomes Exploratory measurement of preeclampsia-related maternal biomarkers (sFLT-1, sEng, PIGF, ET-1 and VCAM-1) Others	31 October 2018	Recruiting	https://www.anzctr.org.au/Trial/Registration/TrialReview.aspx?id=374798 (accessed on 4 October 2021)
ACTRN12618001755224	Can esomeprazole improve outcomes in women at high risk of pre-eclampsia? The ESPRESSO Study	Multi-centre, double blind, randomised, placebo-controlled superiority trial.	Pregnant women screened at 11 + 0 to 13 + 6 weeks gestation and at high risk (>1%) of pre-eclampsia	Esomeprazole 40 mg oral tablet once a day prior to 16 weeks gestation and continuing until delivery of pregnancy. Aspirin 150 mg oral tablet at night commencing prior to 16 weeks gestation and continuing until 36 weeks gestation as a background therapy	Primary outcome measures: Mean arterial pressure, measured by 24-h ambulatory blood pressure at 36 weeks of gestation Secondary outcome measures: Circulating sFlt-1, sEN, PLGF, sFlt-1/PLGF ratio concentrations at 36 weeks of gestation Weight of the baby, agar score, neonatal hospital discharge	18 April 2019	Recruiting	https://www.anzctr.org.au/Trial/Registration/TrialReview.aspx?id=375343 (accessed on 4 October 2021)

## Data Availability

The data that support the findings of this study are available from the corresponding author upon reasonable request.

## Supplementary Materials

The following are available online at https://www.mdpi.com/article/10.3390/jcm11164675/s1: Supplementary Table S1: PRISMA and MOOSE checklists. Table S2: Complete search strategy. Table S3: List of articles excluded with reasons during the full-text screening.
