# Supplementary material for: Proton Pump Inhibitors Use and Risk of Preeclampsia: A Meta-Analysis
Source: J Clin Med. 2022 Aug 10;11(16):4675. doi: 10.3390/jcm11164675 (PMC9410414; doi:10.3390/jcm11164675)
Supplement: Supplementary file 1 [file jcm-11-04675-s001.zip › jcm-1789811-supplementary.pdf]

Supplementary Table S1 (PRISMA 2020 Checklist)

Section and Topic	Item #	Checklist item	Location where item is reported
**TITLE**			
Title	1	Identify the report as a systematic review.	1
**ABSTRACT**			
Abstract	2	See the PRISMA 2020 for Abstracts checklist.	2
**INTRODUCTION**			
Rationale	3	Describe the rationale for the review in the context of existing knowledge.	3
Objectives	4	Provide an explicit statement of the objective(s) or question(s) the review addresses.	3
**METHODS**			
Eligibility criteria	5	Specify the inclusion and exclusion criteria for the review and how studies were grouped for the syntheses.	4
Information sources	6	Specify all databases, registers, websites, organisations, reference lists and other sources searched or consulted to identify studies. Specify the date when each source was last searched or consulted.	4
Search strategy	7	Present the full search strategies for all databases, registers and websites, including any filters and limits used.	Supplement
Selection process	8	Specify the methods used to decide whether a study met the inclusion criteria of the review, including how many reviewers screened each record and each report retrieved, whether they worked independently, and if applicable, details of automation tools used in the process.	4-5
Data collection process	9	Specify the methods used to collect data from reports, including how many reviewers collected data from each report, whether they worked independently, any processes for obtaining or confirming data from study investigators, and if applicable, details of automation tools used in the process.	5
Data items	10a	List and define all outcomes for which data were sought. Specify whether all results that were compatible with each outcome domain in each study were sought (e.g. for all measures, time points, analyses), and if not, the methods used to decide which results to collect.	5
	10b	List and define all other variables for which data were sought (e.g. participant and intervention characteristics, funding sources). Describe any assumptions made about any missing or unclear information.	5
Study risk of bias assessment	11	Specify the methods used to assess risk of bias in the included studies, including details of the tool(s) used, how many reviewers assessed each study and whether they worked independently, and if applicable, details of automation tools used in the process.	5
Effect measures	12	Specify for each outcome the effect measure(s) (e.g. risk ratio, mean difference) used in the synthesis or presentation of results.	5
Synthesis methods	13a	Describe the processes used to decide which studies were eligible for each synthesis (e.g. tabulating the study intervention characteristics and comparing against the planned groups for each synthesis (item #5)).	5
	13b	Describe any methods required to prepare the data for presentation or synthesis, such as handling of missing summary statistics, or data	5-6

Section and Topic	Item #	Checklist item	Location where item is reported
		conversions.	
	13c	Describe any methods used to tabulate or visually display results of individual studies and syntheses.	5-6
	13d	Describe any methods used to synthesize results and provide a rationale for the choice(s). If meta-analysis was performed, describe the model(s), method(s) to identify the presence and extent of statistical heterogeneity, and software package(s) used.	5-6
	13e	Describe any methods used to explore possible causes of heterogeneity among study results (e.g. subgroup analysis, meta-regression).	5-6
	13f	Describe any sensitivity analyses conducted to assess robustness of the synthesized results.	6
Reporting bias assessment	14	Describe any methods used to assess risk of bias due to missing results in a synthesis (arising from reporting biases).	NA
Certainty assessment	15	Describe any methods used to assess certainty (or confidence) in the body of evidence for an outcome.	6
**RESULTS**			
Study selection	16a	Describe the results of the search and selection process, from the number of records identified in the search to the number of studies included in the review, ideally using a flow diagram.	6, Fig.1
	16b	Cite studies that might appear to meet the inclusion criteria, but which were excluded, and explain why they were excluded.	NA
Study characteristics	17	Cite each included study and present its characteristics.	6-7, Table 1
Risk of bias in studies	18	Present assessments of risk of bias for each included study.	Table 2
Results of individual studies	19	For all outcomes, present, for each study: (a) summary statistics for each group (where appropriate) and (b) an effect estimate and its precision (e.g. confidence/credible interval), ideally using structured tables or plots.	7, Fig.2-5
Results of syntheses	20a	For each synthesis, briefly summarise the characteristics and risk of bias among contributing studies.	7
	20b	Present results of all statistical syntheses conducted. If meta-analysis was done, present for each the summary estimate and its precision (e.g. confidence/credible interval) and measures of statistical heterogeneity. If comparing groups, describe the direction of the effect.	7-8
	20c	Present results of all investigations of possible causes of heterogeneity among study results.	7
	20d	Present results of all sensitivity analyses conducted to assess the robustness of the synthesized results.	8
Reporting biases	21	Present assessments of risk of bias due to missing results (arising from reporting biases) for each synthesis assessed.	NA
Certainty of evidence	22	Present assessments of certainty (or confidence) in the body of evidence for each outcome assessed.	9-10, Table 3

Section and Topic	Item #	Checklist item	Location where item is reported
**DISCUSSION**			
Discussion	23a	Provide a general interpretation of the results in the context of other evidence.	10-11
	23b	Discuss any limitations of the evidence included in the review.	11
	23c	Discuss any limitations of the review processes used.	11
	23d	Discuss implications of the results for practice, policy, and future research.	11
**OTHER INFORMATION**			
Registration and protocol	24a	Provide registration information for the review, including register name and registration number, or state that the review was not registered.	4
	24b	Indicate where the review protocol can be accessed, or state that a protocol was not prepared.	medRxiv
	24c	Describe and explain any amendments to information provided at registration or in the protocol.	NA
Support	25	Describe sources of financial or non-financial support for the review, and the role of the funders or sponsors in the review.	Title page
Competing interests	26	Declare any competing interests of review authors.	Title page
Availability of data, code and other materials	27	Report which of the following are publicly available and where they can be found: template data collection forms; data extracted from included studies; data used for all analyses; analytic code; any other materials used in the review.	Title page

From: Page MJ, McKenzie JE, Bossuyt PM, Boutron I, Hoffmann TC, Mulrow CD, et al. The PRISMA 2020 statement: an updated guideline for reporting systematic reviews. BMJ 2021;372:n71. doi: 10.1136/bmj.n71

Supplementary Table S1 (MOOSE Checklist)

Item No	Recommendation	Reported on Page No
Reporting of background should include		
1	Problem definition	1
2	Hypothesis statement	2
3	Description of study outcome(s)	2
4	Type of exposure or intervention used	2
5	Type of study designs used	2-3
6	Study population	2-3
Reporting of search strategy should include		
7	Qualifications of searchers (eg, librarians and investigators)	4
8	Search strategy, including time period included in the synthesis and key words	4, supplement
9	Effort to include all available studies, including contact with authors	4
10	Databases and registries searched	4
11	Search software used, name and version, including special features used (eg, explosion)	3-4
12	Use of hand searching (eg, reference lists of obtained articles)	4
13	List of citations located and those excluded, including justification	6, supplement
14	Method of addressing articles published in languages other than English	NA
15	Method of handling abstracts and unpublished studies	4
16	Description of any contact with authors	Not Required
Reporting of methods should include		
17	Description of relevance or appropriateness of studies assembled for assessing the hypothesis to be tested	4-5
18	Rationale for the selection and coding of data (eg, sound clinical principles or convenience)	5
19	Documentation of how data were classified and coded (eg, multiple raters, blinding and interrater reliability)	5
20	Assessment of confounding (eg, comparability of cases and controls in studies where appropriate)	5
21	Assessment of study quality, including blinding of quality assessors, stratification or regression on possible predictors of study results	5
22	Assessment of heterogeneity	5-6
23	Description of statistical methods (eg, complete description of fixed or random effects models, justification of whether the chosen models account for predictors of study results, dose-response models, or cumulative meta-analysis) in sufficient detail to be replicated	5-6

24	Provision of appropriate tables and graphics	6
Reporting of results should include		
25	Graphic summarizing individual study estimates and overall estimate	7, Fig.2-5
26	Table giving descriptive information for each study included	6, Table 1
27	Results of sensitivity testing (eg, subgroup analysis)	10
28	Indication of statistical uncertainty of findings	9-10, Table 3

Reporting of discussion should include		
29	Quantitative assessment of bias (eg, publication bias)	NA
30	Justification for exclusion (eg, exclusion of non-English language citations)	NA
31	Assessment of quality of included studies	Table 2
Reporting of conclusions should include		
32	Consideration of alternative explanations for observed results	10-11
33	Generalization of the conclusions (ie, appropriate for the data presented and within the domain of the literature review)	11
34	Guidelines for future research	11-12
35	Disclosure of funding source	Title page

From: Stroup DF, Berlin JA, Morton SC, et al, for the Meta-analysis Of Observational Studies in Epidemiology (MOOSE) Group. Meta-analysis of Observational Studes in Epidemiology. A Proposal for Reporting. *JAMA*. 2000;283(15):2008-2012. doi: 10.1001/jama.283.15.2008.

Supplementary Table S2 (Search Strategy)

MEDLINE(R) ALL <1946 to September 13, 2021> (Ovid)

Search was conducted on 14th September 2021 at 11:25 am (CET).

#	search string	# of results
**1**	exp Proton Pump Inhibitors/	19853
**2**	exp 2-Pyridinylmethylsulfinylbenzimidazoles/	11480
**3**	((proton adj2 pump adj2 inhibitor*) or PPI or PPIs).tw,kf,kw.	32778
**4**	(hydrogen potassium adenosine triphosphatase inhibitor* or hydrogen potassium atpase inhibitor*).tw,kf,kw.	2
**5**	(dexilant or dexlansoprazol or dexlansoprazole or kapidex).tw,kf,kw.	131
**6**	(alenia or escz or esofag or esomeprazol or esomeprazole or esotrex or nexiam or nexium or perprazole or sompraz or vimovo).tw,kf,kw.	1721
**7**	(agopton or bamalite or inhibitol or lansol or lan#oprazol or lan#oprazole or lanzor or levant or levolansoprazole or lupizole or monolitum or ogast or ogastro or opiren or prevacid or prezal or promeco or pro ulco or takepron or ulpax or zoton).tw,kf,kw.	3035
**8**	(epirazole or losec or lomac or madiprazole or medoprazole or miracid or ocid or omeprazol or omeprazole or omeprazon or omeprazen or omez or prilosec or prisolec or rapinex or zegerid).tw,kf,kw.	8710
**9**	(astropan or controloc or inipomp or pantecta or pantodac or pantoloc or pantop or pantopan or pantopra#ol or pantopra#ole or pantor or pantotab or pantozol or pantozole or protium or protonix or somac or ulcepraz or zentro or zurcal or zurcale).tw,kf,kw.	2171
**10**	(aciphex or dexrabeprazole or nzole-D or pariet or pariprazole or rabecid or rabeloc or rabeprazol or rabeprazole or rabicip or rablet or zechin).tw,kf,kw.	1260
**11**	(ilaprazole or timoprazole?).tw,kf,kw.	88
**12**	or/1-11	46916
**13**	Pre-Eclampsia/ or Eclampsia/	35183
**14**	(eclamp* or preeclamp*).tw,kf,kw.	39180
**15**	(EPH adj1 (complex* or tox?emi* or gestos#s)).tw,kf,kw.	478
**16**	edema proteinuria hypertension gestos#s.tw,kf,kw.	13
**17**	((hypertens* or high blood pressure) adj3 (pregnan* or gestation*)).tw,kf,kw.	16463
**18**	(pregnan* adj3 tox?emi*).tw,kf,kw.	4829
**19**	or/13-18	59101
**20**	12 and 19	45

Embase 1974 to 2021 September 13

Search was conducted on 14th September 2021 at 11:42 am (CET).

#	search string	# of results
**1**	exp proton pump inhibitor/	85096
**2**	((proton adj2 pump adj2 inhibitor*) or PPI or PPIs).tw,kw.	50766
**3**	(hydrogen potassium adenosine triphosphatase inhibitor* or hydrogen potassium atpase inhibitor*).tw,kw.	4
**4**	(dexilant or dexlansoprazol or dexlansoprazole or kapidex).tw,kw.	309
**5**	(alenia or escz or esofag or esomeprazol or esomeprazole or esotrex or nexiam or nexium or perprazole or sompraz or vimovo).tw,kw.	4217
**6**	(agopton or bamalite or inhibitol or lansol or lan#oprazol or lan#oprazole or lanzor or levant or levolansoprazole or lupizole or monolitum or ogast or ogastro or opiren or prevacid or prezal or promeco or pro ulco or takepron or ulpax or zoton).tw,kw.	5268
**7**	(epirazole or losec or lomac or madiprazole or medoprazole or miracid or ocid or omeprazol or omeprazole or omeprazon or omeprazen or omez or prilosec or prisolec or rapinex or zegerid).tw,kw.	14483
**8**	(astropan or controloc or inipomp or pantecta or pantodac or pantoloc or pantop or pantopan or pantopra#ol or pantopra#ole or pantor or pantotab or pantozol or pantozole or protium or protonix or somac or ulcepraz or zentro or zurcal or zurcale).tw,kw.	4872
**9**	(aciphex or dexrabeprazole or nzole-D or pariet or pariprazole or rabecid or rabeloc or rabeprazol or rabeprazole or rabicip or rablet or zechin).tw,kw.	2552
**10**	(ilaprazole or timoprazole?).tw,kw.	134
**11**	or/1-10	111731
**12**	exp "eclampsia and preeclampsia"/	64020
**13**	(eclamp* or preeclamp*).tw,kw.	56850
**14**	(EPH adj1 (complex* or tox?emi* or gestos#s)).tw,kw.	554
**15**	edema proteinuria hypertension gestos#s.tw,kw.	9
**16**	((hypertens* or high blood pressure) adj3 (pregnan* or gestation*)).tw,kw.	24000
**17**	(pregnan* adj3 tox?emi*).tw,kw.	1631
**18**	or/12-17	83758
**19**	11 and 18	171

Emcare 1995 to 2021 Week 36 (Ovid)

Search was conducted on 14th September 2021 at 11:50 pm (CET).

#	search string	# of results
**1**	exp proton pump inhibitor/	19692
**2**	((proton adj2 pump adj2 inhibitor*) or PPI or PPIs).tw,kw.	8311
**3**	(hydrogen potassium adenosine triphosphatase inhibitor* or hydrogen potassium atpase inhibitor*).tw,kw.	0
**4**	(dexilant or dexlansoprazol or dexlansoprazole or kapidex).tw,kw.	71
**5**	(alenia or escz or esofag or esomeprazol or esomeprazole or esotrex or nexiam or nexium or perprazole or sompraz or vimovo).tw,kw.	731
**6**	(agopton or bamalite or inhibitol or lansol or lan#oprazol or lan#oprazole or lanzor or levant or levolansoprazole or lupizole or monolitum or ogast or ogastro or opiren or prevacid or prezal or promeco or pro ulco or takepron or ulpax or zoton).tw,kw.	950
**7**	(epirazole or losec or lomac or madiprazole or medoprazole or miracid or ocid or omeprazol or omeprazole or omeprazon or omeprazen or omez or prilosec or prisolec or rapinex or zegerid).tw,kw.	2020
**8**	(astropan or controloc or inipomp or pantecta or pantodac or pantoloc or pantop or pantopan or pantopra#ol or pantopra#ole or pantor or pantotab or pantozol or pantozole or protium or protonix or somac or ulcepraz or zentro or zurcal or zurcale).tw,kw.	753
**9**	(aciphex or dexrabeprazole or nzole-D or pariet or pariprazole or rabecid or rabeloc or rabeprazol or rabeprazole or rabicip or rablet or zechin).tw,kw.	362
**10**	(ilaprazole or timoprazole?).tw,kw.	10
**11**	or/1-10	23709
**12**	exp "eclampsia and preeclampsia"/	15147
**13**	(eclamp* or preeclamp*).tw,kw.	11986
**14**	(EPH adj1 (complex* or tox?emi* or gestos#s)).tw,kw.	3
**15**	edema proteinuria hypertension gestos#s.tw,kw.	0
**16**	((hypertens* or high blood pressure) adj3 (pregnan* or gestation*)).tw,kw.	4857
**17**	(pregnan* adj3 tox?emi*).tw,kw.	57
**18**	or/12-17	18625
**19**	11 and 18	56

CINAHL with Full Text (EBSCO)

Search modes Boolean/Phrase

Search was conducted on 14th September 2021 at 12:17 pm (CET).

#	search string	# of results
**1**	(MH "Proton Pump Inhibitors+")	5648
**2**	TI ((proton N1 pump N1 inhibitor*) OR PPI OR PPIs) OR AB ((proton N1 pump N1 inhibitor*) OR PPI OR PPIs)	6129
**3**	TI ("hydrogen potassium adenosine triphosphatase inhibitor*" OR "hydrogen potassium atpase inhibitor*") OR AB ("hydrogen potassium adenosine triphosphatase inhibitor*" OR "hydrogen potassium atpase inhibitor*")	0
**4**	TI (dexilant OR dexlansoprazol OR dexlansoprazole OR kapidex) OR AB (dexilant OR dexlansoprazol OR dexlansoprazole OR kapidex)	43
**5**	TI (alenia OR escz OR esofag OR esomeprazol OR esomeprazole OR esotrex OR nexiam OR nexium OR perprazole OR sompraz OR vimovo) OR AB (alenia OR escz OR esofag OR esomeprazol OR esomeprazole OR esotrex OR nexiam OR nexium OR perprazole OR sompraz OR vimovo)	467
**6**	TI (agopton OR bamalite OR inhibitol OR lansol OR lan?oprazol OR lan?oprazole OR lanzor OR levant OR levolansoprazole OR lupizole OR monolitum OR ogast OR ogastro OR opiren OR prevacid OR prezal OR promeco OR "pro ulco" OR takepron OR ulpax OR zoton) OR AB (agopton OR bamalite OR inhibitol OR lansol OR lan?oprazol OR lan?oprazole OR lanzor OR levant OR levolansoprazole OR lupizole OR monolitum OR ogast OR ogastro OR opiren OR prevacid OR prezal OR promeco OR "pro ulco" OR takepron OR ulpax OR zoton)	428
**7**	TI (epirazole OR losec OR lomac OR madiprazole OR medoprazole OR miracid OR ocid OR omeprazol OR omeprazole OR omeprazon OR omeprazen OR omez OR prilosec OR prisolec OR rapinex OR zegerid) OR AB (epirazole OR losec OR lomac OR madiprazole OR medoprazole OR miracid OR ocid OR omeprazol OR omeprazole OR omeprazon OR omeprazen OR omez OR prilosec OR prisolec OR rapinex OR zegerid)	1159
**8**	TI (astropan OR controloc OR inipomp OR pantecta OR pantodac OR pantoloc OR pantop OR pantopan OR pantopra?ol OR pantopra?ole OR pantor OR pantotab OR pantozol OR pantozole OR protium OR protonix OR somac OR ulcepraz OR zentro OR zurcal OR zurcale) OR AB (astropan OR controloc OR inipomp OR pantecta OR pantodac OR pantoloc OR pantop OR pantopan OR pantopra?ol OR pantopra?ole OR pantor OR pantotab OR pantozol OR pantozole OR protium OR protonix OR somac OR ulcepraz OR zentro OR zurcal OR zurcale)	443
**9**	TI (aciphex OR dexrabeprazole OR "nzole-D" OR pariet OR pariprazole OR rabecid OR rabeloc OR rabeprazol OR rabeprazole OR rabicip OR rablet OR zechin) OR AB (aciphex OR dexrabeprazole OR "nzole-D" OR pariet OR pariprazole OR rabecid OR rabeloc OR rabeprazol OR rabeprazole OR rabicip OR rablet OR zechin)	213
**10**	TI (ilaprazole OR timoprazole#) OR AB (ilaprazole OR timoprazole#)	16
**11**	S1 OR S2 OR S3 OR S4 OR S5 OR S6 OR S7 OR S8 OR S9 OR S10	9735
**12**	(MH "Eclampsia+")	9839
**13**	TI (eclamp* OR preeclamp*) OR AB (eclamp* OR preeclamp*)	13378
**14**	TI (EPH N0 (complex* OR tox#emi* OR gestos?s)) OR AB (EPH N0 (complex* OR tox#emi* OR gestos?s))	0

**15**	TI ("edema proteinuria hypertension gestos?s") OR AB ("edema proteinuria hypertension gestos?s")	0
**16**	TI ((hypertens* OR "high blood pressure") N2 (pregnan* OR gestation*)) OR AB ((hypertens* OR "high blood pressure") N2 (pregnan* OR gestation*))	5333
**17**	TI (pregnan* N2 tox#emi*) OR AB (pregnan* N2 tox#emi*)	34
**18**	S12 OR S13 OR S14 OR S15 OR S16 OR S17	18297
**19**	S11 AND S18	30

Web of Science Core Collection

Indexes=SCI-EXPANDED, SSCI, A&HCI, CPCI-S, CPCI-SSH, BKCI-S, BKCI-SSH, ESCI, CCR-EXPANDED, IC Timespan=All years

Search was conducted on 14th September 2021 at 13:45 am (CET).

#	search string	# of results
**1**	TOPIC: ((proton near/1 pump near/1 inhibitor*) or PPI or PPIs)	41479
**2**	TOPIC: ((“hydrogen potassium adenosine triphosphatase inhibitor*” or “hydrogen potassium atpase inhibitor*”))	3
**3**	TOPIC: (“dexilant” or “dexlansoprazol” or “dexlansoprazole” or “kapidex”)	203
**4**	TOPIC: (“alenia” or “escz” or “esofag” or “esomeprazol” or “esomeprazole” or “esotrex” or “nexiam” or “nexium” or “perprazole” or “sompraz” or “vimovo”)	3456
**5**	TOPIC: (“agopton” or “bamalite” or “inhibitol” or “lansol” or lan?oprazol or lan?oprazole or “lanzor” or “levant” or “levolansoprazole” or “lupizole” or “monolitum” or “ogast” or “ogastro” or “opiren” or “prevacid” or “prezal” or “promeco” or “pro ulco” or “takepron” or “ulpax” or “zoton”)	8488
**6**	TOPIC: (“epirazole” or “losec” or “lomac” or “madiprazole” or “medoprazole” or “miracid” or “ocid” or “omeprazol” or “omeprazole” or “omeprazon” or “omeprazen” or “omez” or “prilosec” or “prisolec” or “rapinex” or “zegerid”)	15379
**7**	TOPIC: (“astropan” or “controloc” or “inipomp” or “pantecta” or “pantodac” or “pantoloc” or “pantop” or “pantopan” or pantopra?ol or pantopra?ole or “pantor” or “pantotab” or “pantozol” or “pantozole” or “protium” or “protonix” or “somac” or “ulcepraz” or “zentro” or “zurcal” or “zurcale”)	4209
**8**	TOPIC: (“aciphex” or “dexrabeprazole” or “nzole-D” or “pariet” or “pariprazole” or “rabecid” or “rabeloc” or “rabeprazol” or “rabeprazole” or “rabicip” or “rablet” or “zechin”)	1915
**9**	TOPIC: (“ilaprazole” or timoprazole$)	107
**10**	#1 OR #2 OR #3 OR #4 OR #5 OR #6 OR #7 OR #8 OR #9	61192
**11**	TOPIC: (eclamp* or preeclamp*)	47434
**12**	TOPIC: (EPH near/0 (complex* or tox$emi* or gestos?s)	248
**13**	TOPIC: (“edema proteinuria hypertension gestos?s”)	4
**14**	TOPIC: (hypertens* or “high blood pressure”) near/2 (pregnan* or gestation*)	16810
**15**	TOPIC: (pregnan* near/2 tox$emi*)	1406
**16**	#11 OR #12 OR #13 OR #14 OR #15	56875
**17**	#10 AND #16	57

Scopus

Search was conducted on 14th September 2021 at 14:05–14:10 pm (CET).

#	search string	# of results
**1**	TITLE-ABS-KEY ((proton W/1 pump W/1 inhibitor*) OR ppi OR ppis)	64340
**2**	TITLE-ABS-KEY (("hydrogen potassium adenosine triphosphatase inhibitor*") OR ("hydrogen potassium atpase inhibitor*"))	4
**3**	TITLE-ABS-KEY ("dexilant" OR "dexlansoprazol" OR "dexlansoprazole" OR "kapidex")	383
**4**	TITLE-ABS-KEY ("alenia" OR "escz" OR "esofag" OR "esomeprazol" OR "esomeprazole" OR "esotrex" OR "nexiam" OR "nexium" OR "perprazole" OR "sompraz" OR "vimovo")	9318
**5**	TITLE-ABS-KEY ("agopton" OR "bamalite" OR "inhibitol" OR "lansol" OR lan?oprazol OR lan?oprazole OR "lanzor" OR "levant" OR "levolansoprazole" OR "lupizole" OR "monolitum" OR "ogast" OR "ogastro" OR "opiren" OR "prevacid" OR "prezal" OR "promeco" OR "pro ulco" OR "takepron" OR "ulpax" OR "zoton")	15609
**6**	TITLE-ABS-KEY ("epirazole" OR "losec" OR "lomac" OR "madiprazole" OR "medoprazole" OR "miracid" OR "ocid" OR "omeprazol" OR "omeprazole" OR "omeprazon" OR "omeprazen" OR "omez" OR "prilosec" OR "prisolec" OR "rapinex" OR "zegerid")	34512
**7**	TITLE-ABS-KEY ("astropan" OR "controloc" OR "inipomp" OR "pantecta" OR "pantodac" OR "pantoloc" OR "pantop" OR "pantopan" OR pantopra?ol OR pantopra?ole OR "pantor" OR "pantotab" OR "pantozol" OR "pantozole" OR "protium" OR "protonix" OR "somac" OR "ulcepraz" OR "zentro" OR "zurcal" OR "zurcale")	11472
**8**	TITLE-ABS-KEY ("aciphex" OR "dexrabeprazole" OR "nzole-D" OR "pariet" OR "pariprazole" OR "rabecid" OR "rabeloc" OR "rabeprazol" OR "rabeprazole" OR "rabicip" OR "rablet" OR "zechin")	5150
**9**	TITLE-ABS-KEY ("ilaprazole" OR timoprazole*)	264
**10**	#1 OR #2 OR #3 OR #4 OR #5 OR #6 OR #7 OR #8 OR #9	106604
**11**	TITLE-ABS-KEY (eclamp* OR preeclamp*)	63100
**12**	TITLE-ABS-KEY (eph W/0 (complex* OR toxemi* OR toxaemi* OR gestos?s))	772
**13**	TITLE-ABS-KEY ("edema proteinuria hypertension gestos?s")	14
**14**	TITLE-ABS-KEY ((hypertens* OR "high blood pressure") W/2 (pregnan* OR gestation*))	19508
**15**	TITLE-ABS-KEY (pregnan* W/2 (toxemi* OR toxaemi*))	11375
**16**	#11 OR #12 OR #13 OR #14 OR #15	78801
**17**	#10 AND #16	143

ProQuest Dissertations & Theses Global

Search was conducted on 14th September 2021 at 14:30 am (CET).

#	search string	# of results
**1**	TIABSU((proton n/1 pump n/1 inhibitor*) or PPI or PPIs)	1678
**2**	TIABSU("hydrogen potassium adenosine triphosphatase inhibitor*" or "hydrogen potassium atpase inhibitor*")	0
**3**	TIABSU(dexilant or dexlansoprazol or dexlansoprazole or kapidex)	0
**4**	TIABSU(alenia or escz or esofag or esomeprazol or esomeprazole or esotrex or nexiam or nexium or perprazole or sompraz or vimovo)	28
**5**	TIABSU(agopton or bamalite or inhibitol or lansol or lan?oprazol or lan?oprazole or lanzor or levant or levolansoprazole or lupizole or monolitum or ogast or ogastro or opiren or prevacid or prezal or promeco or "pro ulco" or takepron or ulpax or zoton)	809
**6**	TIABSU(epirazole or losec or lomac or madiprazole or medoprazole or miracid or ocid or omeprazol or omeprazole or omeprazon or omeprazen or omez or prilosec or prisolec or rapinex or zegerid)	236
**7**	TIABSU(astropan or controloc or inipomp or pantecta or pantodac or pantoloc or pantop or pantopan or pantopra?ol or pantopra?ole or pantor or pantotab or pantozol or pantozole or protium or protonix or somac or ulcepraz or zentro or zurcal or zurcale)	96
**8**	TIABSU(aciphex or dexrabeprazole or "nzole-D" or pariet or pariprazole or rabecid or rabeloc or rabeprazol or rabeprazole or rabicip or rablet or zechin)	8
**9**	TIABSU(ilaprazole or timoprazole?)	1
**10**	S1 OR S2 OR S3 OR S4 OR S5 OR S6 OR S7 OR S8 OR S9	2758
**11**	TIABSU(eclamp* or preeclamp*)	1291
**12**	TIABSU(EPH n/0 (complex* or toxaemi* OR toxemi* or gestos?s))	1
**13**	TIABSU("edema proteinuria hypertension gestos?s")	0
**14**	TIABSU(hypertens* n/2 (pregnan* or gestation*)) or ("high blood pressure" n/2 (pregnan* or gestation*))	1390
**15**	TIABSU(pregnan* n/2 (toxaemi* OR toxemi*))	67
**16**	S11 OR S12 OR S13 OR S14 OR S15	2382
**17**	S10 AND S16	2

WHO International Clinical Trials Registry Platform (ICTRP)

Search was conducted on 14th September 2021 at 15:05–15:20 pm (CET).

#	search string	# of results
**1**	preeclampsia OR eclampsia OR pre-eclampsia OR EPH complex OR EPH toxemia OR EPH toxaemia OR EPH gestosis OR EPH-complex OR EPH-toxemia OR EPH-toxaemia OR EPH-gestosis OR edema proteinuria hypertension gestosis OR hypertension pregnancy OR gestational hypertension OR toxemia pregnancy OR toxaemia pregnancy**– in Field Title**	
**2**	inhibitor of proton pump OR proton pump inhibitor OR PPI OR dexlansoprazole OR esomeprazole OR lansoprazole OR omeprazole OR pantoprazole OR rabeprazole OR ilaprazole OR timoprazole **– in Field Intervention**	
**3**	Recruitment status – ALL	
**4**	1 AND 2 AND 3	13

Search was conducted on 14th September 2021 at 15:25–15:30 pm (CET).

#	search string	# of results
**1**	preeclampsia OR eclampsia OR pre-eclampsia OR EPH complex OR EPH toxemia OR EPH toxaemia OR EPH gestosis OR EPH-complex OR EPH-toxemia OR EPH-toxaemia OR EPH-gestosis OR gestational hypertension OR toxemia pregnancy OR toxaemia pregnancy **– in Field Condition**	
**2**	inhibitor of proton pump OR proton pump inhibitor OR PPI OR dexlansoprazole OR esomeprazole OR lansoprazole OR omeprazole OR pantoprazole OR rabeprazole OR ilaprazole OR timoprazole **– in Field Intervention**	
**3**	Recruitment status – ALL	
**4**	1 AND 2 AND 3	13

Search was conducted on 14th September 2021 at 15:35–15:40 pm (CET).

#	search string	# of results
**1**	preeclampsia OR eclampsia OR pre-eclampsia OR EPH complex OR EPH toxemia OR EPH toxaemia OR EPH gestosis OR EPH-complex OR EPH-toxemia OR EPH-toxaemia OR EPH-gestosis OR gestational hypertension OR toxemia pregnancy OR toxaemia pregnancy **– in Field Condition**	
**2**	inhibitor of proton pump OR proton pump inhibitor OR PPI OR dexlansoprazole OR esomeprazole OR lansoprazole OR omeprazole OR pantoprazole OR rabeprazole OR ilaprazole OR timoprazole **– in Field Title**	
**3**	Recruitment status – ALL	
**4**	1 AND 2 AND 3	15

ClinicalTrials.gov
Search was conducted on 14th September 2021 at 14:50–14:55 am (CET).

#	search string	# of results
**1**	preeclampsia OR eclampsia OR "EPH complex" OR "EPH toxemia" OR "EPH toxaemia"OR "EPH gestosis" OR "edema proteinuria hypertension gestosis" OR "hypertension pregnancy" OR "gestational hypertension" OR "toxemia pregnancy" OR "toxaemia pregnancy" **– in Field Condition or disease**	
**2**	"inhibitor of proton pump" OR "proton pump inhibitor" OR PPI OR dexlansoprazole OR esomeprazole OR lansoprazole OR omeprazole OR pantoprazole OR rabeprazole OR ilaprazole OR timoprazole **– in Field Other terms**	
**3**	1 AND 2	7

Supplementary Table S3 (List of excluded articles)

Author, year	Title	Reason for exclusion
Colvin et al. 2017 [1]	Dispensing patterns and pregnancy outcomes in women dispensed proton pump inhibitors during pregnancy	Outcome not of interest
Saleh et al. 2017 [2]	Low soluble FMS-like tyrosine kinase-1, endoglin, and endothelin-1 levels in women with confirmed or suspected preeclampsia using proton pump inhibitors	Outcome not of interest
Cluver et al. 2018 [3]	Esomeprazole to treat women with preterm preeclampsia: a randomized placebo controlled trial	Outcome not of interest
Ferdousmakan et al. 2021 [4]	Medication Safety in Obstetrics and Gynecology Ward in Jayanagar General Hospital, Bangalore, India	Outcome not of interest
Sun et al. 2021 [5]	Megalin, Proton Pump Inhibitors and the Renin-Angiotensin System in Healthy and Pre-Eclamptic Placentas	Outcome not of interest
Onda et al. 2017 [6]	Proton pump inhibitors decrease soluble fms-like tyrosine kinase-1 and soluble endoglin secretion, decrease hypertension, and rescue endothelial dysfunction.	Outcome not of interest
Kaitu'u-Lino et al. 2018 [7]	Combining metformin and esomeprazole is additive in reducing sFlt-1 secretion and decreasing endothelial dysfunction–implications for treating preeclampsia	Outcome not of interest
Groom et al. 2018 [8]	The role of aspirin, heparin, and other interventions in the prevention and treatment of fetal growth restriction	Outcome not of interest
Binder et al. 2020 [9]	Esomeprazole and sulfasalazine in combination additively reduce sFlt-1 secretion and diminish endothelial dysfunction: potential for a combination treatment for preeclampsia	Outcome not of interest
Cluver et al. 2018 [10]	Th Pre-eclampsia Intervention with Esomeprazole trial (PIE trial)	Study design not of interest
Onda et al. 2015 [11]	Proton pump inhibitors for treatment of preeclampsia	Study design not of interest
Hodge et al. 2007 [12]	Alterations in drug disposition during pregnancy: Implications for drug therapy	Study design not of interest

References

1. Colvin L, Slack-Smith L, Stanley F, Bower C. 104. Dispensing Patterns and Pregnancy Outcomes in Women Dispensed Proton Pump Inhibitors during Pregnancy. Pharmacoepidemiology and Drug Safety. 2011;20:45-6.
2. Saleh L, Samantar R, Garrelds IM, van den Meiracker AH, Visser W, Danser AJ. Low soluble Fms-like tyrosine kinase-1, endoglin, and endothelin-1 levels in women with confirmed or suspected preeclampsia using proton pump inhibitors. Hypertension. 2017 Sep;70(3):594-600.
3. Cluver CA, Hannan NJ, van Papendorp E, Hiscock R, Beard S, Mol BW, Theron GB, Hall DR, Decloedt EH, Stander M, Adams KT. Esomeprazole to treat women with preterm preeclampsia: a randomized placebo-controlled trial. American journal of obstetrics and gynecology. 2018 Oct 1;219(4):388-e1.
4. Ferdousmakan S, KB N, Etesami M. Medication Safety in Obstetrics and Gynecology Ward in Jayanagar General Hospital, Bangalore, India. Pakistan Journal of Medical and Health Sciences. 2021;15(5):1270-5.
5. Sun Y, Tan L, Neuman RI, Broekhuizen M, Schoenmakers S, Lu X, Danser AH. Megalin, Proton Pump Inhibitors and the Renin–Angiotensin System in Healthy and Pre-Eclamptic Placentas. International Journal of Molecular Sciences. 2021 Jan;22(14):7407.
6. Onda K, Tong S, Beard S, Binder N, Muto M, Senadheera SN, Parry L, Dilworth M, Renshall L, Brownfoot F, Hastie R. Proton pump inhibitors decrease soluble fms-like tyrosine kinase-1 and soluble endoglin secretion, decrease hypertension, and rescue endothelial dysfunction. Hypertension. 2017;69(3):457-68.
7. Kaitu'u-Lino TU, Brownfoot FC, Beard S, Cannon P, Hastie R, Nguyen TV, Binder NK, Tong S, Hannan NJ. Combining metformin and esomeprazole is additive in reducing sFlt-1 secretion and decreasing endothelial dysfunction–implications for treating preeclampsia. PLoS One. 2018;13(2):e0188845.
8. Groom KM, David AL. The role of aspirin, heparin, and other interventions in the prevention and treatment of fetal growth restriction. American journal of obstetrics and gynecology. 2018;218(2):S829-40.
9. Binder NK, Brownfoot FC, Beard S, Cannon P, Nguyen TV, Tong S, Tu'uhevaha J, Hannan NJ. Esomeprazole and sulfasalazine in combination additively reduce sFlt-1 secretion and diminish endothelial dysfunction: Potential for a combination treatment for preeclampsia. Pregnancy Hypertension. 2020;22:86-92.
10. Cluver C, Hannan N, van Papendorp E, Hiscock R, Mol B, Theron G, Hall D, Rensburg M, Beard S, Schubert P, Walker S. 34: Th Pre-eclampsia Intervention with Esomeprazole trial (PIE trial). American Journal of Obstetrics & Gynecology. 2018;218(1):S26-7.
11. Onda K, Hannan N, Beard S, Binder N, Brownfoot F, Kaitu'u-Lino TU, Tuohey L, Hastie R, Tong S. [6-OR]: Proton pump inhibitors for treatment of preeclampsia. Pregnancy Hypertension: An International Journal of Women's Cardiovascular Health. 2015;5(1):3.
12. Hodge LS, Tracy TS. Alterations in drug disposition during pregnancy: implications for drug therapy. Expert opinion on drug metabolism & toxicology. 2007;3(4):557-71.
